# Supplementary material for: Dopaminergic Modulation of Short‐Term Associative Memory in Caenorhabditis elegans
Source: J Neurochem. 2025 Aug 19;169(8):e70200. doi: 10.1111/jnc.70200 (PMC12362331; doi:10.1111/jnc.70200)
Supplement: Supplementary file 2 — Data S2: jnc70200‐sup‐0002‐Supinfo02.pdf. [file JNC-169-0-s002.pdf]

Supplementary material relating to

Dopaminergic Modulation of Short-Term Associative Memory in *Caenorhabditis elegans*

Anna McMillen[1], Caitlin Minervini[1], Renee Green[1], Michaela E. Johnson[1], Radwan Ansaar[1], Yee Lian Chew[1]

1 - Flinders Health and Medical Research Institute, College of Medicine and Public Health, Flinders University, Bedford Park 5042, South Australia

* - to whom correspondence should be addressed: yeelian.chew@flinders.edu.au

Supplementary Figure legends

Figure S1: Dopamine receptors *dop-1*, *dop-2* and *dop-3* act redundantly to modulate short-term memory. Learning indices calculated for naïve/untrained worms and conditioned animals at 0, 0.5-, 1-, 1.5- and 2-hours post-conditioning, for chemotaxis data shown in Figure 2: **A)** *dop-1* mutants, **B)** *dop-2* mutants, **C)** *dop-3* mutants, **D)** *dop-1;dop-2* double mutants, **E)** *dop-1;dop-3* double mutants. Graphs show 3-4 biological replicates: each data point represents one biological replicate (which includes four technical replicates). Each technical replicate consists of 50-250 worms. Statistical analysis: a one-way ANOVA with Tukey's multiple comparisons test was performed to compare between genotypes for all post-conditioning time points. Error bars = mean ± SD. p-values are represented by **** ≤ 0.0001; *** ≤ 0.001, ** ≤ 0.01; * ≤ 0.05; ns = non-significant.

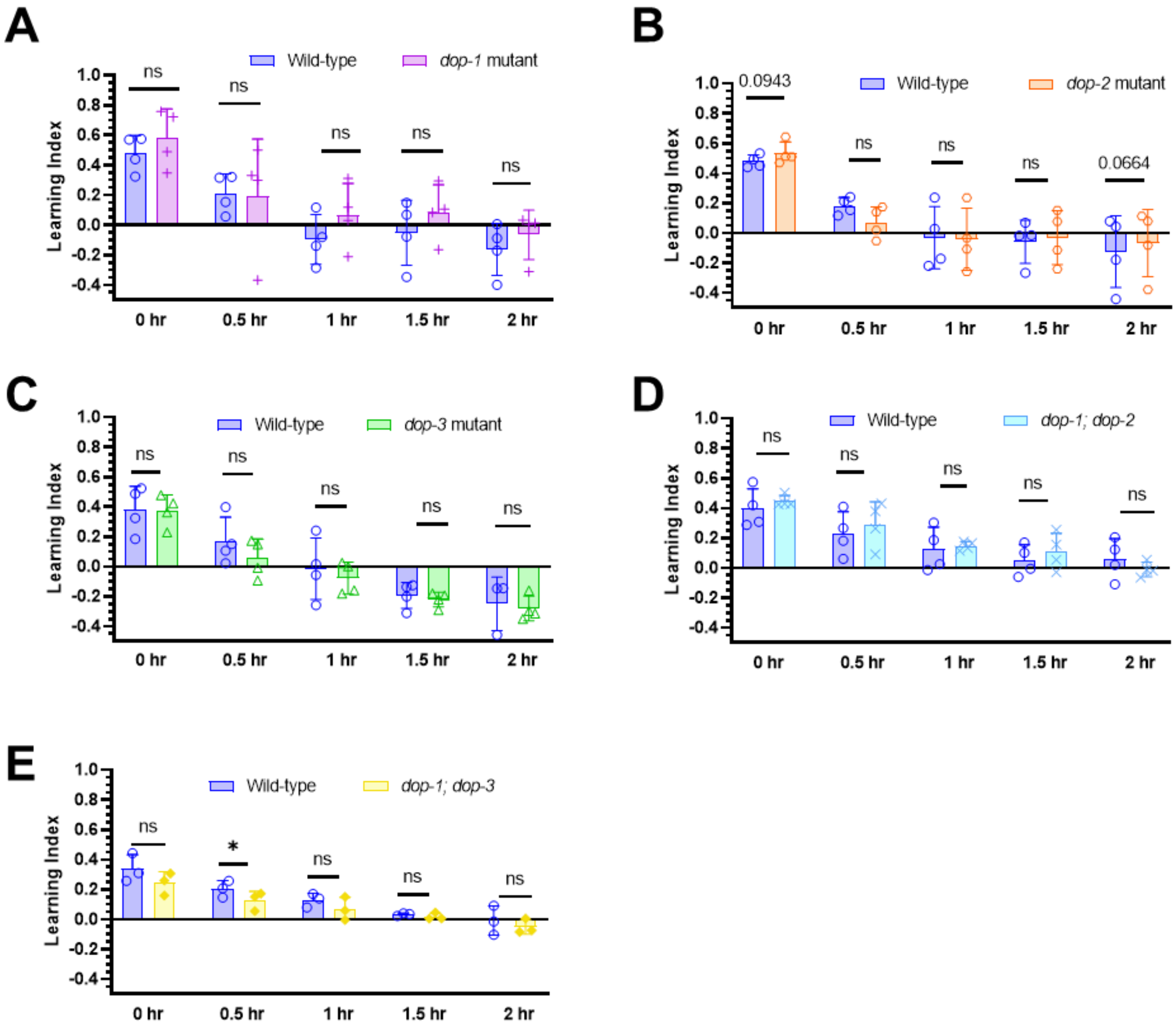
A
Wild-type
dop-1 mutant
Learning Index
ns
0 hr
0.5 hr
1 hr
1.5 hr
2 hr
B
Wild-type
dop-2 mutant
0.0943
0.0664
C
Wild-type
dop-3 mutant
D
Wild-type
dop-1; dop-2
E
Wild-type
dop-1; dop-3
*

Figure S1
